# Optimizing Bi_2_O_3_ and TiO_2_ to achieve the maximum non-linear electrical property of ZnO low voltage varistor

**DOI:** 10.1186/1752-153X-7-137

**Published:** 2013-08-10

**Authors:** Yadollah Abdollahi, Azmi Zakaria, Raba’ah Syahidah Aziz, Siti Norazilah Ahmad Tamili, Khamirul Amin Matori, Nuraine Mariana Mohd Shahrani, Nurhidayati Mohd Sidek, Masoumeh Dorraj, Seyedehmaryam Moosavi

**Affiliations:** 1 Material Synthesis and Characterization Laboratory, Institute of Advanced Technology, Universiti Putra Malaysia, 43400 UPM, Serdang, Selangor, Malaysia; 2 Department of Physics, Faculty Science, Universiti Putra Malaysia, 43400 UPM, Serdang, Selangor, Malaysia

**Keywords:** Optimization, ZnO-varistor, Modeling, RSM, Bi_2_O_3_, TiO_2_

## Abstract

**Background:**

In fabrication of ZnO-based low voltage varistor, Bi_2_O_3_ and TiO_2_ have been used as former and grain growth enhancer factors respectively. Therefore, the molar ratio of the factors is quit important in the fabrication. In this paper, modeling and optimization of Bi_2_O_3_ and TiO_2_ was carried out by response surface methodology to achieve maximized electrical properties. The fabrication was planned by central composite design using two variables and one response. To obtain actual responses, the design was performed in laboratory by the conventional methods of ceramics fabrication. The actual responses were fitted into a valid second order algebraic polynomial equation. Then the quadratic model was suggested by response surface methodology. The model was validated by analysis of variance which provided several evidences such as high F-value (153.6), very low P-value (<0.0001), adjusted R-squared (0.985) and predicted R-squared (0.947). Moreover, the lack of fit was not significant which means the model was significant.

**Results:**

The model tracked the optimum of the additives in the design by using three dimension surface plots. In the optimum condition, the molars ratio of Bi_2_O_3_ and TiO_2_ were obtained in a surface area around 1.25 point that maximized the nonlinear coefficient around 20 point. Moreover, the model predicted the optimum amount of the additives in desirable condition. In this case, the condition included minimum standard error (0.35) and maximum nonlinearity (20.03), while molar ratio of Bi_2_O_3_ (1.24 mol%) and TiO_2_ (1.27 mol%) was in range. The condition as a solution was tested by further experiments for confirmation. As the experimental results showed, the obtained value of the non-linearity, 21.6, was quite close to the predicted model.

**Conclusion:**

Response surface methodology has been successful for modeling and optimizing the additives such as Bi_2_O_3_ and TiO_2_ of ZnO-based low voltage varistor to achieve maximized non-linearity properties.

## Background

Varistors are nonlinear electro-devices with a ceramics microstructure that are used as protectors in distribution and energy transmission lines against voltage surge [1]. In the past four decades, varistors based on ZnO and SnO_2_ have attracted attention because of their excellent non-ohmic behavior and low leakage currents [2,3]. However, ZnO-based varistor has been demanded along with the development of very-large-scale integration electronics because it exhibits high nonlinear current–voltage (I-V) characteristics in lower voltage ranges [4,5]. The non-linearity is expressed by I = KV^α^ where K is a constant, and ‘α’ is nonlinear coefficient (alpha) [6]. The alpha originates from microstructure of the varistor ceramics which is composed of conductive n-type ZnO grains and small amount of a few metal oxide additives such as Bi_2_O_3_, TiO_2_, Co_3_O_4_, Mn_2_O_3_, Sb_2_O_3_ and Al_2_O_3_. The microstructure is made of ZnO grain surrounded by the melted additives as boundaries [4]. The boundaries contain of Bi-rich intergranular, metal oxide and secondary spinel phase and strictly influences on the alpha [4,6-11]. The role of Bi_2_O_3_, as a former, is quite important since it provides the medium for liquid-phase sintering, enhances the growth of ZnO grains, and finally stables the nonlinear current–voltage characteristics of the varistor [12]. High sintering temperature is necessary for ZnO grain growth despite the fact that at this condition Bi_2_O_3_ tends to evaporate [13]. The melting point of Bi_2_O_3_ is 825°C, and the eutectic temperature of ZnO-Bi_2_O_3_ is only 740°C, thus a liquid phase is formed in the ZnO-Bi_2_O_3_ specimens below 800°C. As soon as the eutectic liquid is formed, the mass loss starts to increase which indicates the vaporization of Bi_2_O_3_[14]. The sharp lost weight was reported above 1100°C since there was no reported peaks of β-Bi_2_O_3_ at 1300°C [15,16]. On the other hand, TiO_2_ increases reactivity of the Bi_2_O_3_-rich liquid phase with the solid ZnO during sintering process which prevents Bi_2_O_3_ vaporization [13,17-19]. The phase equilibrium and the temperature of liquid-phase formation are defined by the TiO_2_/Bi_2_O_3_ ratio [20]. According to the reports, the effect of TiO_2_ depends on Bi_2_O_3_ that means the additives interact in low-voltage varistor ceramics fabrication. To determine the effect of the interactions on the varistors’ electrical properties, the molar ratios of the additives must simultaneously be considered. To the best of our knowledge, there is no study on the interactions which optimize the ratio of the additives and maximize the alpha. Recently, response surface methodology (RSM) has been accepted for modeling and optimizing of input intractable variables to achieve maximum yield product as output for productive process [21]. RSM is known as a semi-empirical method because the process could be optimized by using experimental results, and a group of mathematical and statistical techniques [22]. In this work, RSM was used for modeling and optimizing of molar ratio of Bi_2_O_3_ and TiO_2_ as additives to achieve the maximum value of the alpha for low voltage varistor. The experiments were designed by central composite design (CCD) to obtain the empirical results (actual). The results were used for regression and fitting process to fine an appropriate model. The model was verified by several statistical techniques such as residual analysis, scaling residuals and prediction error sum of squares (PRESS). The model optimized the input additives and then maximized the alpha as output. In addition, the model predicted the desirable condition including minimum standard error and the maximum alpha which are validated by further experiments. The predicted samples were characterized by X-ray diffractometer (XRD), scanning electron microscope (SEM), variable pressure scanning electron microscope (VPSEM) and Energy-dispersive X-ray (EDX).

## Experimental

### Materials and methods

The commercial chemical, ZnO (99.99%), Bi_2_O_3_ (99.975%), TiO_2_ (99.9%), Sb_2_O_3_ (99.6%), Mn_3_O_4_ (98%), Co_3_O_4_ (99.7%) and Al(NO_3_)_3_ (100% ±2), were provided from Alfa Aesar as starting powders. The powders were weighed according to the experimental design of molar ratios (Table 1). The molar ratios of the powders were mixed, grounded in dry form and then ball milled in acetone for 24 h. During ball milling, agglomeration was controlled by Zirconium oxide balls. After drying in hot oven for 8 h, the mixed powders were grounded and pressed into pellet forms of 10 mm in diameter and 0.70 mm thickness at 200 MPa by a uniaxial presser machine. The disks were sintered in a box furnace (CMTS Model HTS 1400) for holding time of 2 h at 1260°C. The heating and cooling rate were 5°C/min [23]. To determine DC current–voltage (I-V), both of the sintered sample surfaces were coated by silver electrodes. The I-V of the samples was measured by Keithley 236 source meter. The samples were scanned with dc voltage from 0 to 100 V in step size of 2.5 V. The alpha was calculated at J_1_ = 0.1 and J_2_ = 1 mA/cm^2^ by equation (1) as actual responses [6].

(1) $$\alpha =\left({\mathrm{logI}}_{2}-{\mathrm{logI}}_{1}\right)/\left({\mathrm{logV}}_{2}-{\mathrm{logV}}_{1}\right)$$

**Table 1:** Experimental-design contain of the actual variables, and actual response and model predicted values of the alpha

**Run**	**ZnO**	**TiO**_**2**_	**Bi**_**2**_**O**_**3**_	**Co**_**3**_**O**_**4**_	**Mn**_**2**_**O**_**3**_	**Sb**_**2**_**O**_**3**_	**Al(NO**_**3**_**)**_**3**_	**Alpha (Actual)**	**Alpha (Predicted)**
1	96.50	1	1	0.5	0.5	0.5	0.00094	9.3	10.1
2	96.00	1.5	1	0.5	0.5	0.5	0.00094	3.9	3.9
3	96.00	1	1.5	0.5	0.5	0.5	0.00094	6.4	7.4
4	95.50	1.5	1.5	0.5	0.5	0.5	0.00094	10.5	10.6
5	96.35	0.896	1.25	0.5	0.5	0.5	0.00094	9	7.9
6	95.65	1.604	1.25	0.5	0.5	0.5	0.00094	5.6	5.8
7	96.35	1.25	0.896	0.5	0.5	0.5	0.00094	8.2	7.7
8	95.65	1.25	1.604	0.5	0.5	0.5	0.00094	11	10.5
9	96.00	1.25	1.25	0.5	0.5	0.5	0.00094	20	20
10	96.00	1.25	1.25	0.5	0.5	0.5	0.00094	19.6	20
11	96.00	1.25	1.25	0.5	0.5	0.5	0.00094	20.2	20
12	96.00	1.25	1.25	0.5	0.5	0.5	0.00094	20.7	20
13	96.00	1.25	1.25	0.5	0.5	0.5	0.00094	19.4	20

The breakdown voltage (E_b_) was determined by measuring E at J = 0.75 mA/cm^2^ and the leakage current (J_L_) was determined evaluating J at 0.8E_b_ where J (mA/cm^2^) is the current density and E is the electrical field (V/mm). To characterize the microstructure, the both surfaces of samples were polished by aluminum oxide powder. Then, they were etched at 160°C under sintering time with heating and cooling rate, 10°C/min. Phase analysis was conducted using XRD (PANalytical, Philips-X’pert Pro PW3040/60) with CuKα source. The sample were radiated with Ni-filtered CuKα radiation (λ = 1.5428) within the 2θ scan range of 20–80°. Surface morphology and elemental analyses of sintered samples were studied under SEM (JEOL JSM 6400) and VPSEM (LEO 1455) which attached to EDX. The samples was mounted on Al stub using carbon paint and coated by gold layer. Average grains size of the ZnO in the varistor was evaluated by measuring 100 grains in SEMs images.

### Experimental design

The experimental design was carried out by CCD that used Design-Expert software version 8.0.7.1, Stat-Ease Inc., USA [24-26]. CCD is well fixed for fitting a quadratic surface that usually works well for optimization process [27-29]. The variables number, level of variables and number of responses are determined as input of experimental design. In this case, the molar ratio of Bi_2_O_3_ and TiO_2_ was selected as effective variables in vicinity of their optimum while alpha was the response as output. The CCD transformed the variables and the response to the terms of code values (Table 2) because the units and range of variables were different. The coded values spaces are ±1 from the center (0.0) and the star points are usually located ‘α’ distance from the center [29,30]. In the design, there N experiment that includes the factorial points (2^n^), the axial points (2n), and the center points C_o_ or replications as the equation N = 2^n^ + 2n + C_o_ which is 13, 4, 4 and 5 respectively. The replications are used to measure experimental error [31]. As a result, the experimental design is presented as a design matrix with ‘n’ column and ‘N’ row in Table 1. Where, each column corresponds to a particular variable, e.g. *x*_1_ and *x*_2_ which arranged in order to increase factor number from left to right. The rows are experiments runs because each one contains the descriptions of an experiment. Additionally, the design constructs a matrix of actual responses that obtained by the experiments.

**Table 2:** The variables and employed levels in the CCD for ZnO low voltage varistor fabrication

		**Level of variables**		
**Symbol**	**Unit (%)**	**Low (−1)**	**Middle (0)**	**High (+1)**
Bi_2_O_3_ (x_1_)	mol	1.0	1.25	1.5
TiO_2_ (*x*_2_)	mol	1.0	1.25	1.5

### The RSM description

The RSM develops an adequate functional relationship between input variables and interested responses by low-degree approximation of the polynomial models such as the second degree model (Eq. 2) [32].

(2) $$Y=\beta_{o}+\sum_{i=1}^{n}\beta_{i}x_{i}+\sum_{i=1}^{n}\beta_{\mathrm{ii}}x_{i}^{2}+\sum_{i=1}^{n}\sum_{j=i+1}^{n}\beta_{\mathrm{ij}}x_{i}x_{j}+\epsilon$$

where *Y* is the interested response, *β*_*0*_ is a constant term, *β*_*i*_ is the coefficient of the linear terms, *β*_*ii*_ represents the coefficient of the quadratic terms, *β*_*ij*_ is the coefficient of the interaction terms while *x*_i_ are control variables and ‘*ϵ*’ is a random experimental error [31]. For system with two factors, the model is described by equation (3),

(3) $$Y_{1i}=\beta_{0}+\beta_{1}x_{1i}+\beta_{11}x_{1i}^{2}+\beta_{22}x_{2i}^{2}+\beta_{12}x_{1i}x_{2i}+r_{1i}$$

where *Y*_1i_ is the experimental single response, *x*_1_ and *x*_2_ are the coded factors (Table 2), *β*_0_ is the intercept term, *β*_1_ and *β*_2_ are slopes with respect to each of the two factors, *β*_11_ and *β*_22_ are curvature terms, and *β*_12_ is the interaction term. To estimate the *β*’s, the fitting process provides the sufficient data by regression tools [33,34]. In the process, the actual responses are fitted to the polynomial models by sequential model sum of squares (SMSS) [33,34]. The SMSS compares the linear, two-factor interaction (2FI), quadratic and cubic models by using the statistical significance of adding new model terms, step by step in increasing order [35]. To select the provisional model, the lack of fit of those models is compared by minimum p-values and PRESS. The other assessments to select the best provisional model are maximum adjusted R-squared (R_Adj_) and predicted R-squared (R_Pred_) [36]. The p-value is one of the most important evidences which was used to study significant effect of the parameters [33]. In addition, the lack-of-fit test diagnoses how well each terms of the full model fit the data that pillared by statistical parameters such as R_Adj_, R_Pred_ and PRESS [36,37]. Therefore, the provisional model with minimum p-value and PRESS and also maximum R_Adj_, R_Pred_ is selected to investigate in details. The details are provided by using analysis of variance (ANOVA) which contains a collection of terms statistical evidences. The ANOVA determines the significance of intercept, linear, interaction and square terms of the provisional model by using minimum p-value. For more evaluation, the normality of residuals, constant error and residual outlier is checked by various diagnostic plots [38]. The validated model, the relationship between variables and response, is created in coded and actual variables. The model indicates the effect of linear, quadratic and the parameters interactions on the interested response. The effects are presented by estimated coefficients and the related positive and negative signs (+, -). The coefficients are specific weight of the parameters in the model while the signs (+) and (−) operate as synergistic and antagonistic effects on the response [39]. Then the optimization process investigates combination of variables levels that produces the maximum response to a surface area simultaneously. Moreover, the model predicts the yield of product in specific condition such as individual standard error, the range of variables and responses. The prediction could be performed by further experiments.

## Results and discussion

### Modeling

**Figure 1:**
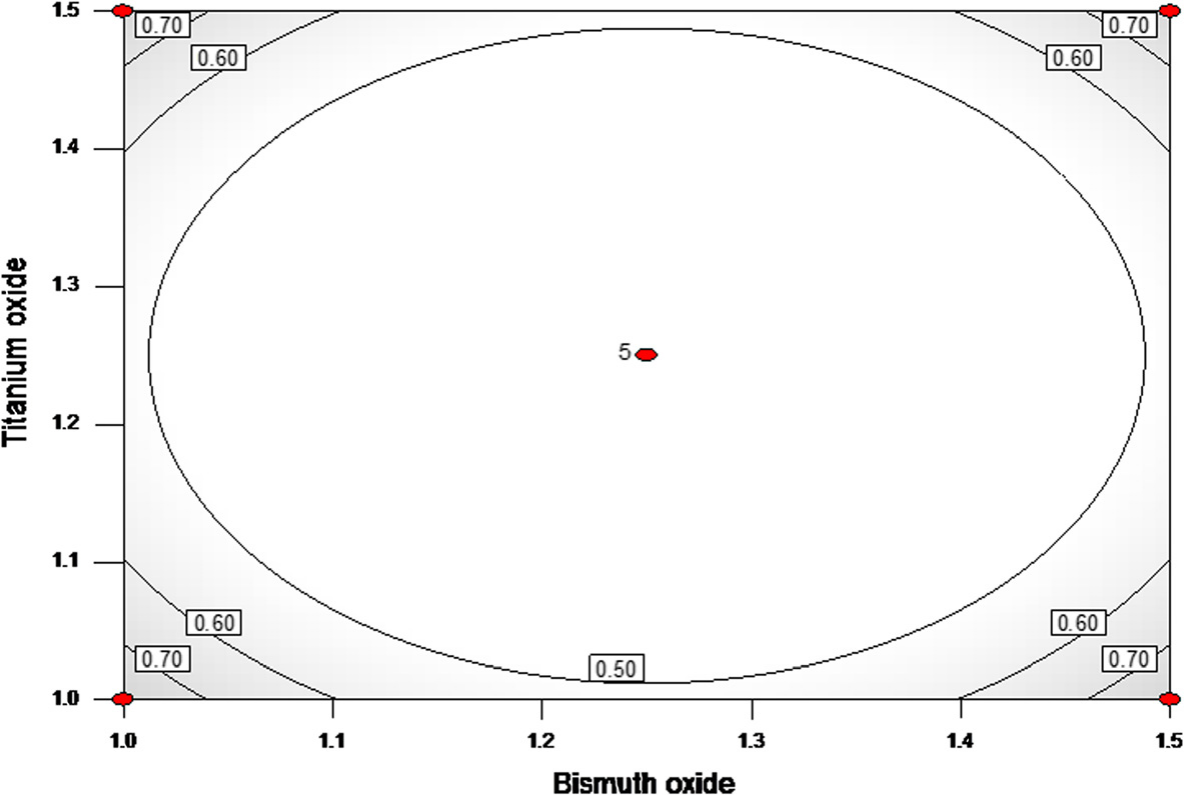
Contour plot of the experimental-design standard error with expanded axes, extrapolated area shaded.

In the fitting process, the residuals are produced from difference between actual and predicted values. Standard error is a great tool to determine the residuals outlier and also the scope of models prediction [40]. Figure 1 indicates the standard error contour plot of the experiment design which displays Bi_2_O_3_ versus TiO_2_ molar ratio. The bright area has relatively low standard error that interested for the modeling and optimization process. However, the darker shading corners represent higher standard errors which are dangerous to extrapolation.

Table 3 indicates that the SMSS compared four models to recommend a proper model [35,36]. As shown, the quadratic model was suggested as the best provisional model. The suggestion based on the lowest standard deviation, p-value and PRESS and the highest R_Adj_, R_Pred_ values. Moreover, the R_Adj_ (0.991) was in reasonable agreement with (<0.20) R_Pred_ (0.985) which confirmed the model sufficiency. Therefore, the authors selected the suggested model to investigate in details by using ANOVA.

**Table 3:** **The sequential model fitting summary for the actual responses which shows statistics conformation of the regression process**, **DF is degree of freedom**

**Source**	**Sum of squares**	**DF**	**Mean square**	**F-Value**	**p**-**value**	**Remark**
**Sequential model sum of squares**						
Mean vs Total	2064.18	1	2064.18	-	-	
Linear vs Mean	12.07	2	6.04	0.13	0.8818	
2FI vs Linear	22.31	1	22.31	0.44	0.5216	
Quadratic vs 2FI	447.11	2	223.55	356.58	< 0.0001	Suggested
Cubic vs Quadratic	1.56	2	0.78	1.38	0.3325	Aliased
Residual	2.83	5	0.57	-	-	
Total	2550.06	13	196.16	-	-	
**Source**	**Sum of squares**	**DF**	**Mean square**	**F-Value**	**p-value**	**Remark**
**Lack of fit tests**						
Linear	472.78	6	78.80	307.73	< 0.0001	
2FI	450.47	5	90.09	351.85	< 0.0001	
Quadratic	3.36	3	1.12	4.38	0.0938	Suggested
Cubic	1.80	1	1.80	7.03	0.0569	Aliased
Pure Error	1.02	4	0.26	-	-	
**Source**	**Std.Dev.**	**R**_**Adj**_	**R**_**Pred**_	**R**	**PRESS**	**Remark**
**Model summary statistics**						
Linear	6.88	0.025	−0.170	−0.571	763.55	
2FI	7.08	0.071	−0.239	−1.195	1066.45	
Quadratic	0.79	0.991	0.985	0.947	25.53	Suggested
Cubic	0.75	0.994	0.986	0.760	116.85	Aliased

Table 4 shows the ANOVA of the provisional model which included the useful statistical evidences about the terms (*x*_1_, *x*_2_, *x*_*1*_*x*_*2*_, *x*_1_^2^ and *x*_2_^2^) in details. As shown, the prob > F of the terms was less than 0.05 which confirmed the high significance of the terms. Moreover, the model’s F-value was 153.6 that indicated great significance for the model. In addition, the very low value of the model p-value confirmed the significance. Futhermore, R-squared (R) provides a measure of how much variability in the observed response values can be explained by the experimental factors and their interactions. In this study, the R (0.991) indicated that the model was capable of accounting for more than 99.1% of the variability in the responses. The R_Adj_ (0.985) was in reasonable agreement with (<0.20) the R_Pred_ (0.947) which confirmed the aptness of the model. The pure errors such as experimental errors were minimal as the lack of fit (0.094) was not significant or the model was fit well. Therefore, ANOVA confirmed the adequacy of the quadratic model that could be used to navigate the design space.

**Table 4:** **Analysis of variance (ANOVA) for response surface quadratic model, MS is mean Square, DF is degree of freedom and SS is sum of squares while *x***_**1 **_**and *x***_**2 **_**introduce in Table **2

**Source**	**SS**	**DF**	**MS**	**F-Value**	**p-value (Prob > F)**	**Suggestion**
Model	481.5	5	96.3	153.6	< 0.0001	significant
x_1_	4.6	1	4.6	7.4	0.0299	
*x*_2_	7.4	1	7.4	11.9	0.0108	
x_1_x_2_	22.3	1	22.3	35.6	0.0006	
x_1_^2^	298.7	1	298.7	476.4	< 0.0001	
x_2_^2^	205.5	1	205.5	327.7	< 0.0001	
Residual	4.4	7	0.6			
Lack of Fit	3.4	3	1.1	4.4	0.0938	not significant
Pure Error	1.0	4	0.3		< 0.0001	
Cor Total	485.9	12			0.0299	
** Std.Dev.**	**R**_**Adj**_	**R**_**Pred**_	**R**^**2**^	**PRESS**	**C.V.%**	**Adeq Precision**
0.792	0.985	0.947	0.991	25.5	6.284	29.9

**Figure 2:**
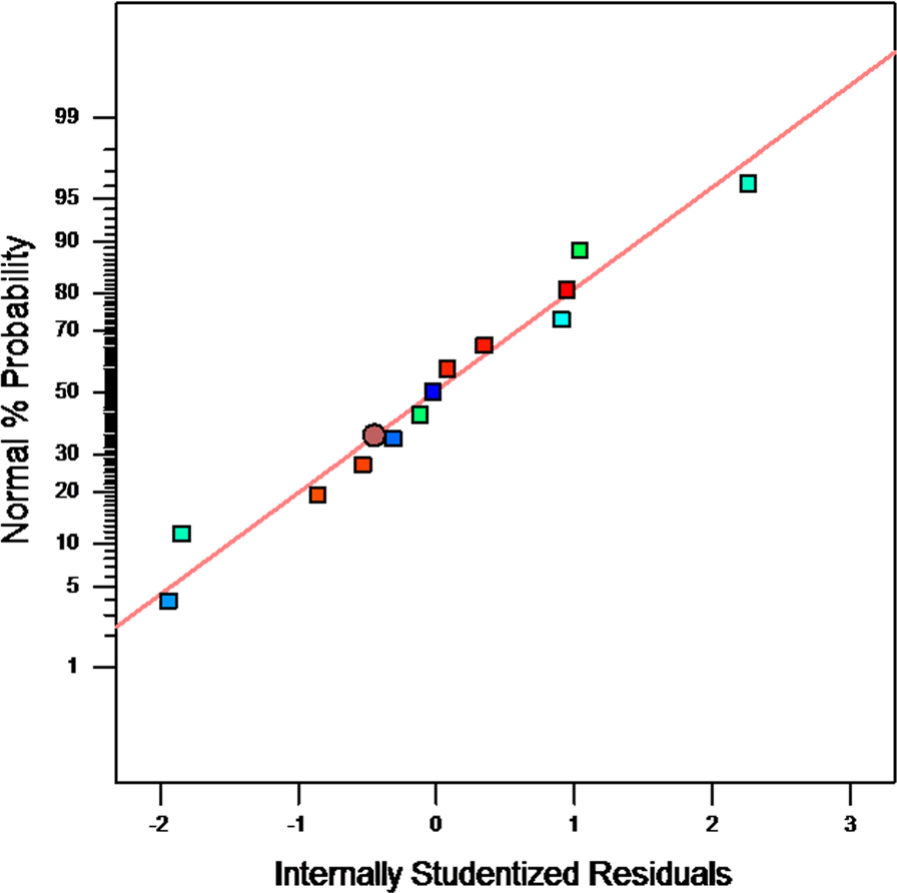
Normal plot of residuals for the whole model.

The normality of residuals, constant error and residual outliers were checked by various diagnostic plots. The normal probability plots of the studentized residuals as one of the most important diagnostic plots, was provided by software default (Figure 2). The plot presents percentage of normal% probability versus internal studentized residuals. The studentized residual is an important technique in the detection of residuals outliers in regressions [41]. As the plot demonstrates, the residuals followed a normal distribution that implies the points follow a straight line.

**Figure 3:**
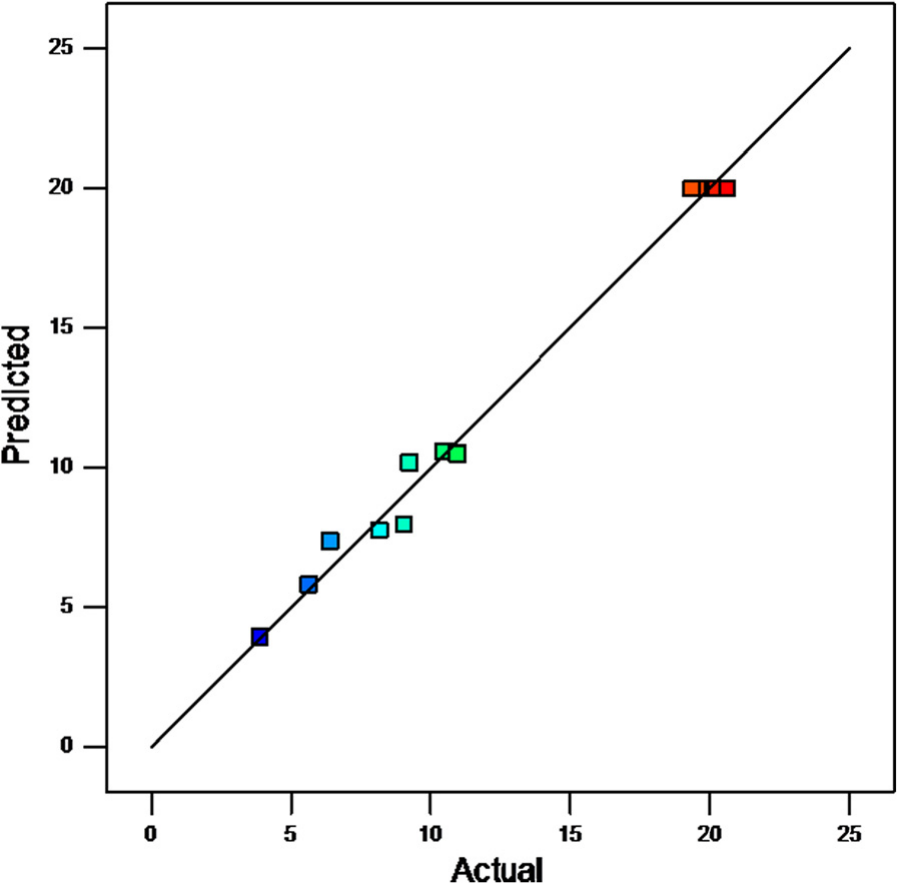
Studentized residuals versus predicted values to check the constant error.

As another diagnosis, Figure 3 illustrates the predicted response values versus the actual response values and detects the values that are not easily predicted by the model. The data points were on the 45 degree line that means the values were not detected. As a result, the diagnosis of residuals reveals that there is no statistical problem in the model.

### The model presentation

The model expresses the relationship between responses of actual variables and the variables themselves. The validated model is presented in actual variables by equation (4),

(4) $$\begin{array}{rl} Y= & -221.67+211.82x_{1}+174.00x_{2}\\ & +37.79x_{1}x_{2}-104.84x_{1}^{2}-86.95x_{2}^{2} \end{array}$$

where the actual values of the variables *x*_1_ and *x*_2_ were shown in Table 2 and Y is the alpha. As shown, the parameters including linear (*x*_1_, *x*_2_), quadratic (*x*_1_^2^, *x*_2_^2^) and interaction (*x*_1_*x*_2_) affected on the interested response. The effects are presented by the individual coefficients and the related signs (+, -) in the model. The coefficients indicate the specific weight of the parameters in the model while the signs are synergistic (+) and antagonistic (−) effects of variables on the response (Y). The weights determine the importance of the parameters roles in the modeling. The model is able to optimize input variables and also approximately predict the response inside of the actual experimental region that confirmed by minimum standard error (Figure 1) [33]. Therefore, the model was used to optimize the molar ratio of Bi_2_O_3_ and TiO_2_ to achieve maximized alpha.

### The model optimization

**Figure 4:**
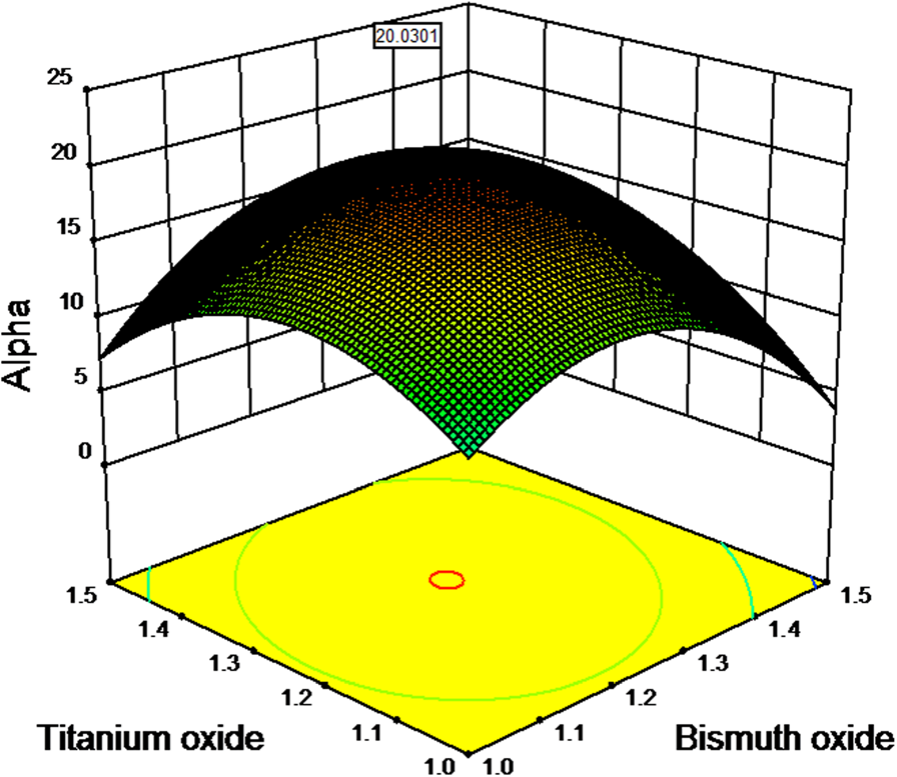
**The effect of Bi**_**2**_**O**_**3 **_**and TiO**_**2 **_**on alpha that simultaneously presented by 3D response surface plot, the maximized alpha was 20.031.**

The model is able to optimize the variables by using canonical response and graphical plots. The canonical responses, local optimums, in terms of the code and actual variables were determined by differentiating the model (Eq. 4) as presented in equations (5 and 6),

(5) $${\left[\partial Y/\partial x_{1}\right]}_{X_{2}}=0$$

(6) $${\left[\partial Y/\partial x_{2}\right]}_{X_{1}}=0$$

where the terms were introduced in Table 2. Therefore, the optimum canonical amount of Bi_2_O_3_ and TiO_2_ were 1.195 and 1.025 respectively. At this optimum, the maximized alpha was 15.03. In fact, the optimization is a kind of the tradition methods, one-variable-at-a-time, because in each case, one of the variables was varied and others were constant. In graphical optimization, the model simultaneously considered the effect of Bi_2_O_3_ and TiO_2_ on the alpha. Figure 4 presents the three dimension response surface (3D) plot for the synergy between Bi_2_O_3_ (1–1.5 mol%) and TiO_2_ (1–1.5 mol%) which is standard error limitation area (Figure 1). As shown, the alpha increased within 1 to 1.25 mol% Bi_2_O_3_ and TiO_2_. However, when the amount of Bi_2_O_3_ and TiO_2_ was increased in excess of the optimum (1.25 mol%), the alpha decreased. Therefore, the optimum was determined in a surface area around 1.25 mol% for the both additives. The maximum alpha was 20.031 at center of the surface which indicated by a flag on top of Figure 4.

The model predicted the maximized alpha in desirable condition of the additives and standard error which facilitated by default of software numerical option. The desirability is an objective function that uses mathematical methods to find the optimum condition [25]. The range of the function is from zero (outside of the limit area) to one (at the goal). The criteria for this case Bi_2_O_3_ (in range), TiO_2_ (in range), standard error (minimized) and alpha (maximized). The suggested solution was Bi_2_O_3_ (1.24 mol%), TiO_2_ (1.27 mol%), standard error (0.35), and alpha 20.03. The desirability of the solution was 0.981 which is close to 100% (at the goal). The solution was performed to confirm the prediction by validated experiment. The validated alpha was determined 21.6 which was quite close to the predicted alpha (20.03). Table 5 illustrated a summary of optimized molar ratios of Bi_2_O_3_ and TiO_2_ and also the related maximized alpha which obtained by canonical, graphical and numerical model optimization method.

**Table 5:** The summary of optimized input variables and obtained maximized photodegradation% by canonical, graphical, numerical methods and validation value of the photodegradation

**Method**	**Bi**_**2**_**O**_**3 **_**(%mol)**	**TiO**_**2 **_**(%mol)**	**Alpha**
Canonical (point)	1.195	1.025	15.03
Graphical (area)	Area around 1.25	Area around 1.25	20.03
Numerical (prediction)	1.24	1.27	20.03
Validated sample	1.24	1.27	21.6

### The validated varistor

**Figure 5:**
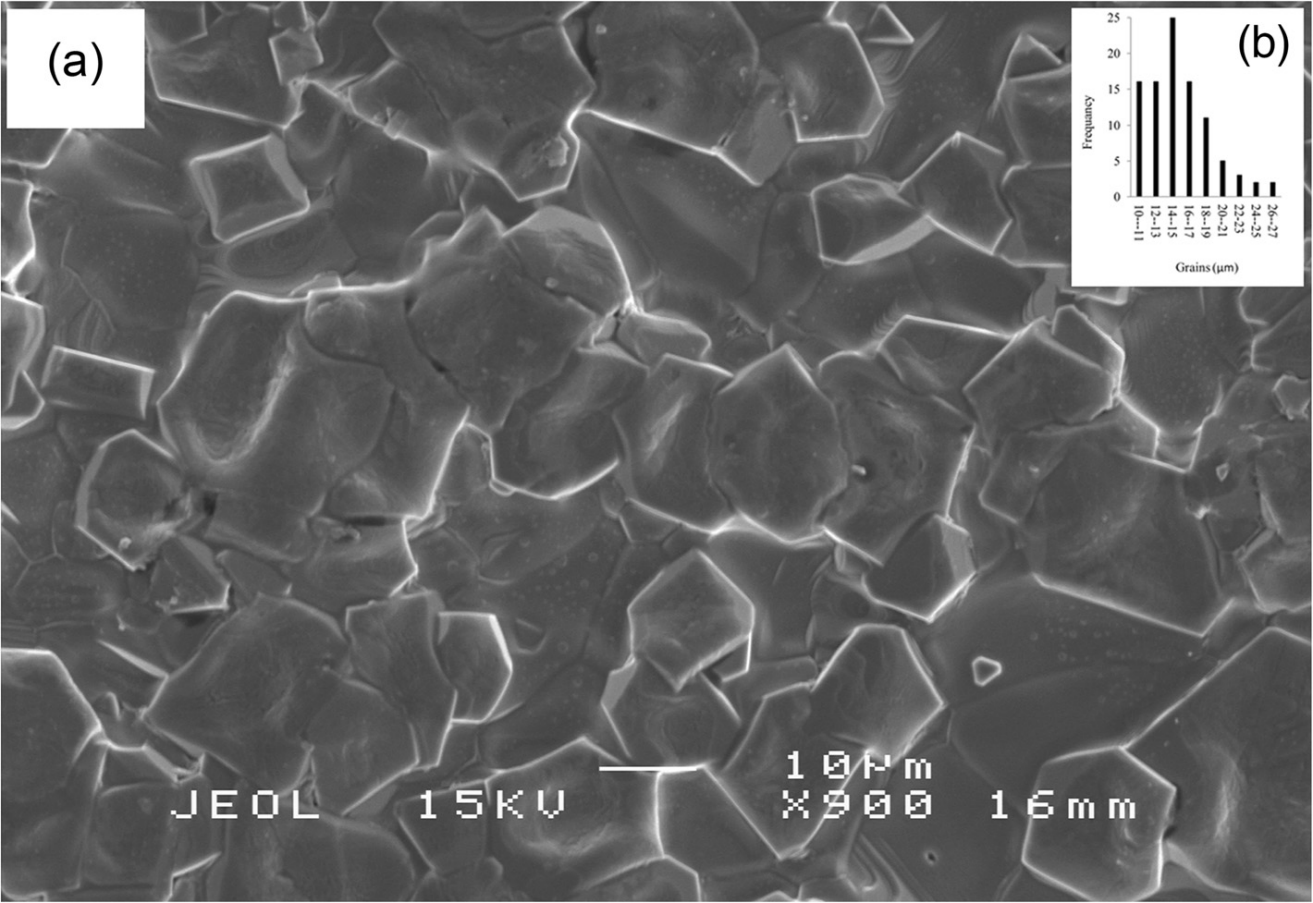
The microstructure of the optimized varistor morphology, (a) SEM (b) distribution of ZnO grain size.

The validated sample was characterized as final varistor for this optimization process. Figure 5 demonstrates the SEM morphology of the sintered ceramic microstructure of the varistor. Figure 5a indicates the great homogeneity of ZnO grain size. The average grain size was 15 μm (Figure 5b).

**Figure 6:**
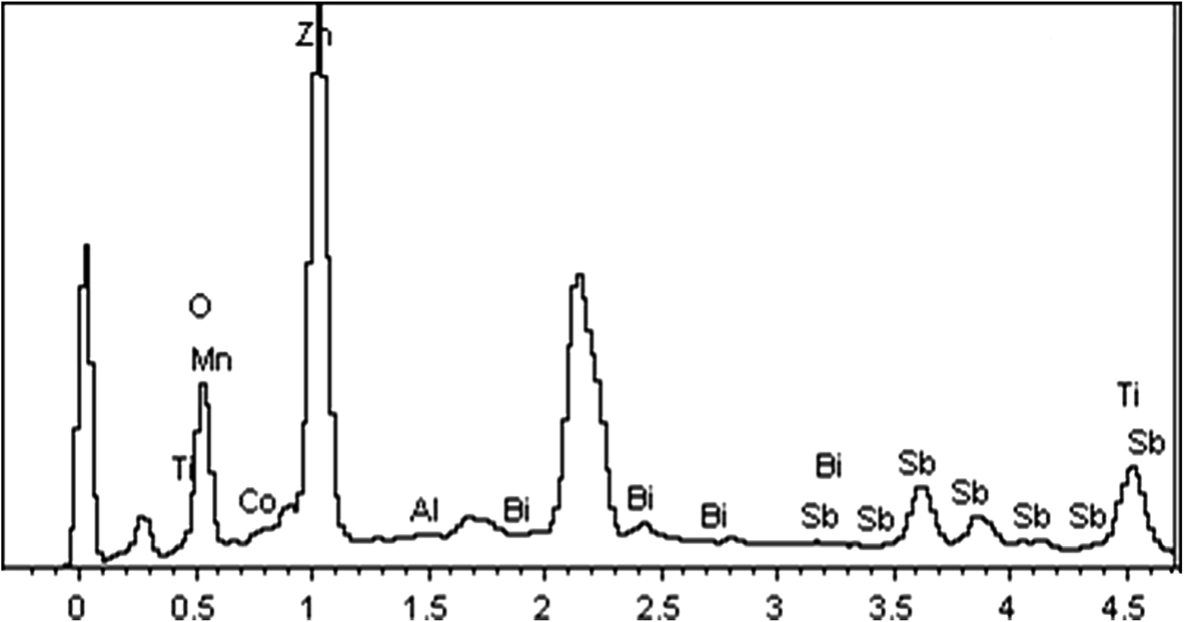
The EDX of etched optimized sample surfaces.

Figure 6 illustrates EDX spectra of a limited area of the etched varistor surfaces composition. As shown all additives particular Bi (1.0 weight%) and Ti (1.17 weight%) were detected in the selected area after sintering process at 1260°C.

**Figure 7:**
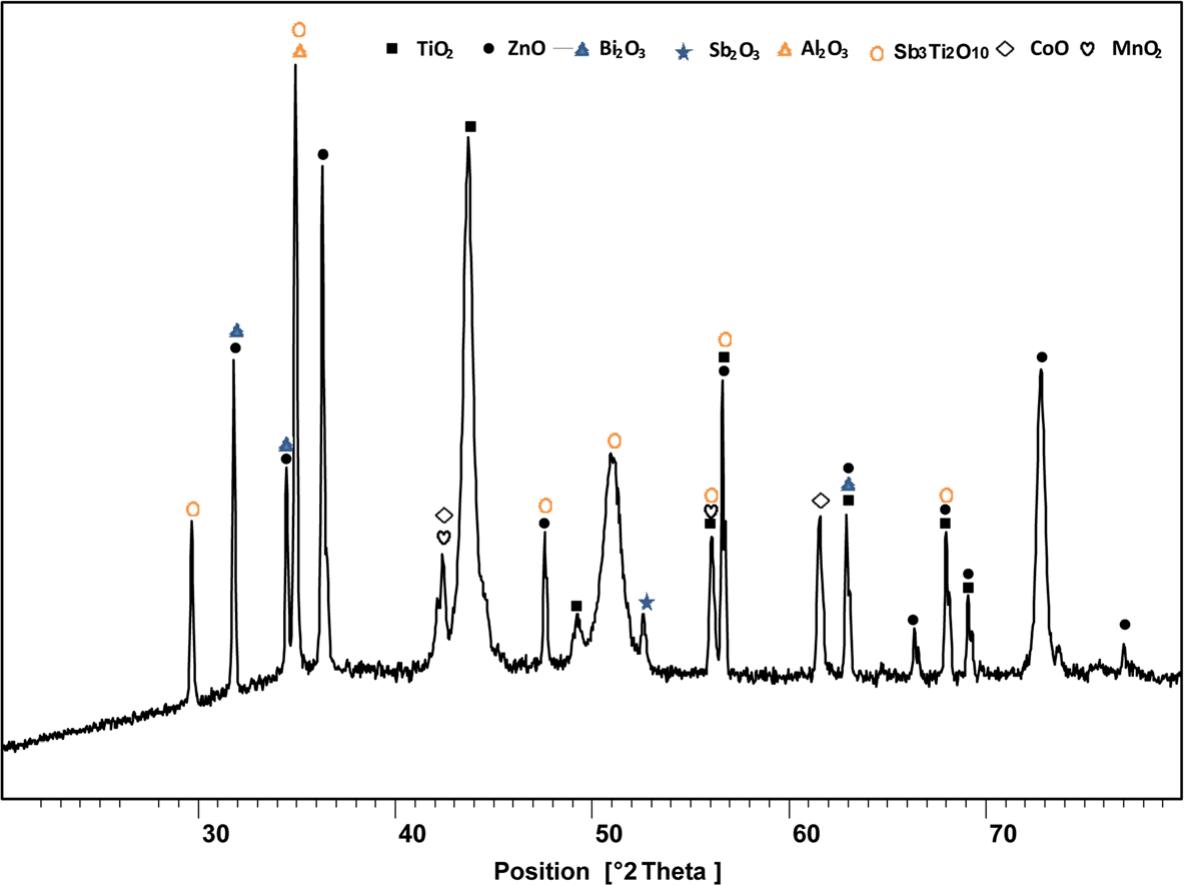
The XRD patterns of optimized varistor sample which include ZnO, additives and spinel.

Figure 7 shows the XRD pattern of the optimized sample which presented three phase of ZnO, spinel and metal oxide of additives. The composites were including ZnO (00-005-0664), Bi_2_O_3_ (00-002-0988), TiO_2_ (01-072-0020), MnO_2_ (00-003-1041), CoO (00-048-1719), Sb_2_O_3_ (01-075-1567), Al_2_O_3_ (00-004-0879) and Sb_3_Ti_2_O_10_ (00-028-0103). The number that mentioned in parenthesis are XRD reference code.

**Figure 8:**
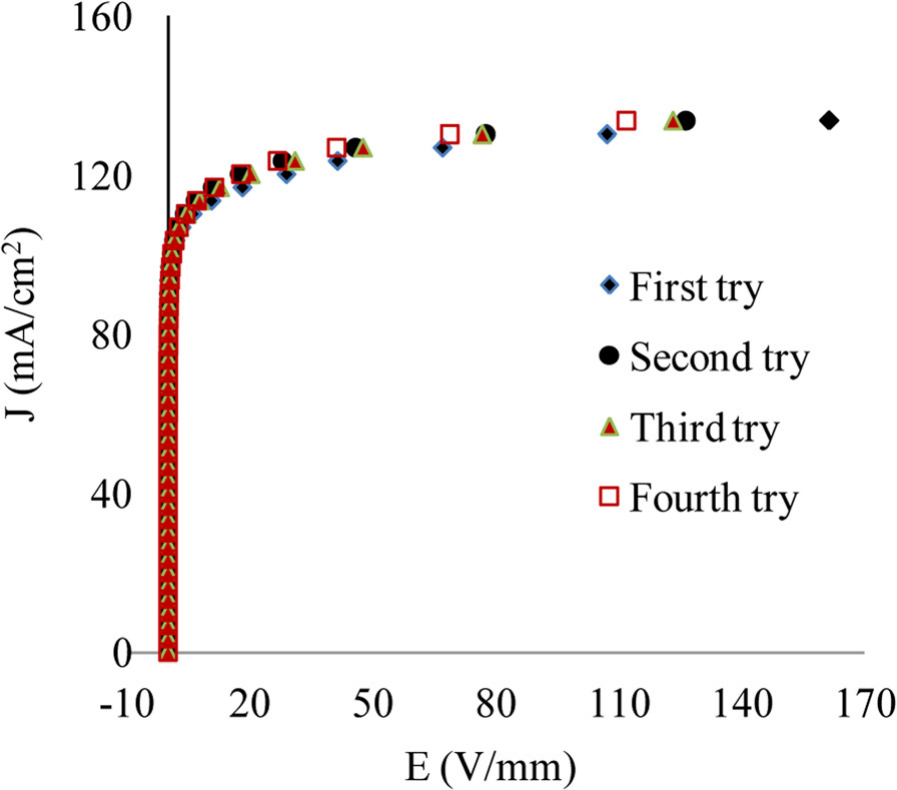
E-J characteristic curves of the optimized samples for first to fourth measurments.

The electrical properties of the varistor were basis of I-V characteristic measurement that shows breakdown voltage was 98 V/mm with non-linear coefficient 21.6. The leakage current was 0.013 mA/cm^2^. The stability of the varistor was measured by alpha recovery after removing the over voltage (Figure 8). As shown the stability was quit significant after fourth over voltage.

## Conclusions

This work reports modeling and optimization of the molar ratio of Bi_2_O_3_ and TiO_2_ by RSM. The fabrication was designed by CCD using two variables and a response. To obtain actual responses, the design was performed in laboratory by conventional fabrication methods. The actual responses were fitted into a quadratic model. The model was validated by ANOVA which provided evidences such as high F-value (153.6), very low p-value (<0.0001), R_adj_ (0.985) and R_Pred_ (0.947). The results of the validation showed the model was significant. The model tracked the optimum of the designed additives by using 3D plots. In the optimum condition, the molars ratio of Bi_2_O_3_ and TiO_2_ were around 1.25 that maximized the alpha value at 20. Moreover, the model suggested a solution to predict the optimum amount of the additives. In this case, the condition of the solution included standard error of 0.35, Bi_2_O_3_ of 1.24, TiO_2_ of 1.27 and alpha of 20.03. The solution was tested by further experiments. As the validation test showed, the obtained value of the alpha (21.6) was very close to the predicted value (20.03). Therefore, RSM was succeeded in modeling of the additives in fabrication of zinc oxide based low voltage varistor to achieve maximum alpha.

## Competing interests

The authors declare that they have no competing interests.

## Authors’ contributions

YA carried out the catalyst design and ligand screening studies. YA, SNA-T, NMM-S and NMS carried out the synthesis, purification and characterization of the compounds. YA carried out the computational experiments. AZ, RSA and KAM conceived of the study, and participated in its design and coordination and helped to draft the manuscript. All authors read and approved the final manuscript.
